# Corneal Topography Analysis of Stromal Corneal Dystrophies

**DOI:** 10.12669/pjms.311.6292

**Published:** 2015

**Authors:** Yusuf Kocluk, Zuleyha Yalniz-Akkaya, Ayse Burcu, Firdevs Ornek

**Affiliations:** 1 Yusuf Kocluk, MD, Ophthalmolgy Clinic, Ministry of Health, Adana Numune Training and Research Hospital, TR01076, Adana, Turkey.; 2 Zuleyha Yalniz-Akkaya, MD, Ophthalmolgy Clinic, Ministry of Health, Ankara Training and Research Hospital, TR06340 Ankara, Turkey.; 3 Ayse Burcu, Associate Professor, Ophthalmolgy Clinic, Ministry of Health, Ankara Training and Research Hospital, TR06340 Ankara, Turkey.; 4 Firdevs Ornek, MD, Ophthalmolgy Clinic, Ministry of Health, Ankara Training and Research Hospital, TR06340 Ankara, Turkey.

**Keywords:** Granular corneal dystrophy, Lattice corneal dystrophy, Macular corneal dystrophy, Pentacam corneal densitometry unit, Scheimpflug imaging

## Abstract

**Objective:**

The aim was to compare the corneal topography and tomography parameters of macular corneal dystrophy (MCD), granular corneal dystrophy (GCD) and lattice corneal dystrophy (LCD) patients obtained by Scheimpflug imaging system.

**Methods:**

The charts, photographs and topography images of patients were reviewed retrospectively. This study included 73 eyes of 73 patients (28 MCD, 20 GCG and 25 LCD patients). Topography images were obtained by Pentacam (Oculus Optikgerate, Wetzlar, Germany). The densitometry readings at the corneal apex were used for the statistics.

**Results:**

The female to male ratio was 13/15 in MCD group, 12/8 in GCD group and 13/12 in LCD group. The mean age median age was 32, 45 and 53 years in MCD, GCD and LCD groups respectively. The groups were similar regarding the gender (p=0.861). The MCD group was significantly younger than the other two groups (p<0.001). The median (minmum-maximum) corneal densities were 100 (100-100), 68 (17-100) and 97 (34-100) Pentacam densitometry units in MCD, GCD and LCD groups respectively. The corneal density at the corneal apex was significantly higher in MCD group than in the other groups (p<0.001). The GCD and LCD groups were statistically similar in terms of density of the corneal apex (p=0.079). In MCD group, corneal thickness at the apex and at the thinnest location was significantly thinner, than in the other groups (p=.002 for thickness at apex between MCD and LCD, and p<.001 for all the remaining comparisons). In MCD group corneal volume was significantly smaller than in the other groups (p<.001 for all comparisons).

**Conclusion:**

Densitometry on Scheimpflug imaging system gives information on the density of corneal opacities.

## INTRODUCTION

The clinical manifestations of the corneal dystrophies depend largely on the layer of the cornea that is affected. The most common corneal stromal dystrophies are macular corneal dystrophy (MCD), granular corneal dystrophy (GCD) and lattice corneal dystrophies (LCD).

MCD is an autosomal recessive disorder characterized by corneal opacities due to intracellular and extracellular deposits within the corneal stroma.^1^ MCD is characterized by a cloudy, dense stroma with gray-white spots. The macular spots have indistinct edges and the intervening stroma becomes unclear. In MCD the early lesions are central and superficial, with involvement of the peripheral cornea and deep stroma over time. Central corneal thinning confirmed by pachymetry has been previously documented.^2^

GCD is a bilateral corneal disorder characterized by the deposition of small, discrete, sharply demarcated, grayish-white opacities in the anterior central stroma. The opacities of GCD can vary in shape, but are usually grouped into three basic morphologic types: drop-shaped, crumb-shaped, and ring-shaped. The overall pattern of deposition is ray or disk-shaped.^3^

LCD usually becomes apparent in both eyes towards the end of the first decade of life, but occasionally it begins in middle life and rarely in infancy. Linear and other shaped opaque areas accumulate particularly within the central corneal stroma, while the peripheral cornea remains relatively transparent. Corneal sensation is often diminished and the interwoven linear opaque filaments have some resemblance to nerves, but may not be evident in all affected members of families with LCD. Both corneas are usually symmetrically involved, but sometimes one cornea remains clear or has discrete rather than linear opacities.^4^

The Pentacam (Oculus Optikgerate, Wetzlar, Germany) is an anterior segment analyzer that implements the Scheimpflug principle in photography to capture slit images and generate a variety of data in a non-contact fashion. The system is equipped with a rotating Scheimpflug camera, and a light source that emits UV-free blue light with a wavelength of 475 nm. All projected slits overlap at the central cornea to increase the accuracy of central data. A single acquisition provides users with color maps of the corneal topography and pachymetry, and elevation maps of the anterior and posterior corneal surfaces.^5^

In this study, we aimed to compare the corneal topography and tomography parameters of MCD, GCD and LCD patients obtained by Scheimpflug imaging.

## METHODS

This retrospective study was conducted according to the tenets of Declaration of Helsinki after the approval of Institutional Review Board. The charts, photographs and topography images of patients were reviewed and patients with MCD, GCD and LCD were identified. This study included 28 eyes of MCD patients, 20 eyes of GCG patients and 25 eyes of LCD patients. Topography images were obtained by Pentacam (Oculus Optikgerate, Wetzlar, Germany) which is capable of generating images of the anterior and posterior surface of the cornea, the iris and the anterior and posterior surface of the lens in a movable virtual eye.^6^ Patients were positioned with a chin rest and head rest and asked to fixate on the centre of the blue slit light. The examiner adjusted the joystick until appropriate alignment was obtained.

All the elevation-based Scheimpflug imaging examinations were performed by well-trained experienced examiners. Spherical body as a reference body, float shape, and 9.00 mm diameter were used at elevation maps in all patients. The patient’s whose Scheimpflug imaging was not possible due to the severity of the dystrophy was excluded from the study. The most reliable images according to quality specification were used for evaluations.

Pentacam provides density measurement using a scale from zero to 100 Pentacam densitometry units. Since vertical meridians can be covered partially by the lids we performed the corneal densitometry evaluation on horizontal Scheimpflug image sections in all the patients. The densitometry readings at the corneal apex were used for the statistics. The evaluated parameters:

- AC Depth: Central anterior chamber depth. It shouldn’t be less than 2.7 mm^3^ to keep the corneal endothelium intact.^7^
- Angle: The average value of anterior chamber volume. An angle less than 25^°^ should alert us to check the patient for angle closure glaucoma.^7^
- CFS Km: Mean curvature power in the central 3 mm cornea front surface (CFS)
- CFS Astig: Astigmatism on CFS
- CFS Q-val (30°): Asphericity in the central 6 mm on CFS. Q-val is considered normal when it falls between 0 and -1.^7^
- CBS Km: Mean curvature power in the central 3 mm cornea back surface (CBS)
- CBS Astig: Astigmatism on CBS
- CBS Q-val (30°): Asphericity in the central 6 mm on CBS
- CV: Corneal volume
- ChV: Chamber volume. Volume less than 100 mm^3^ should alert us to check the patient for angle closure glaucoma.^7^
- DCA: Density of corneal apex
- Kmax: Maximum curvature power on front of cornea. Kmax x and Kmax y coordinates show the position of the Kmax from the apex.
- KPD: Keratometric power deviation. It represents the effect of the back surface of the cornea on the true net power. The normal value at any point should be <+0.75 D. Any value falling between +0.75 D and +1.50 D is doubtful and borderline, but it is not considered significant unless there is a corresponding posterior elevation. Any value more than +1.50 D is an abnormal value, especially if it is in the lower part of the map, or if there is a corresponding elevation at the back elevation map.^7^
- MaxFE: Maximum front elevation at best-fit sphere (BFS) 7.54 float, 9.00 mm diameter. MaxFE x and MaxFE y coordinates show the position of the MaxFE from the apex.
- MaxBE: Maximum back elevation at best-fit sphere (BFS) 6.24 float, 9.00 mm diameter. MaxBE x and MaxBE y coordinates show the position of the MaxBE from the apex.
- PA: Corneal thickness at the apex. The computer considers the apex as the origin of the coordinates, x for the horizontal and y for the vertical. Therefore, zero is displayed in both squares of pachy apex coordinates.^7^
- PCP: Pupil center pachymetry, corneal thickness in the pupil center.
- PD: Pupil diameter
- TLP: Thinnest location pachy in the central 9 mm cornea. TL x and TL y coordinates show the position of the thinnest location from the apex.

***Statistical Analysis: ***Statistical analysis was performed by using SPSS for Windows 16.0 (SPSS Inc. Chicago, USA). The Kolmogorov-Smirnov test was used to check normal distribution of variables. Dependent on the abnormal distribution all of the numerical variables, they were compared using Kruskal Wallis test, and Mann-Whitney U test with Bonferoni correction. The descriptive statistics were expressed as median (minimum-maximum). Qualitative variables were compared with chi-square test. In Kruskal Wallis analyses and Chi-square test a p value less than 0.05 was considered statistically significant. Mann-Whitney U tests with Bonferroni correction was applied in multiple comparisons and p value less than 0.017 (0.05/n, where n is number of comparisons) was considered statistically significant. In our study 3 comparisons (MCD vs. GCD, MCD vs. LCD and GCD vs. LCD) were made.

## RESULTS

The groups were similar regarding the gender (p=0.861) and laterality (p=0.793). The MCD group was significantly younger than the other two groups (p<0.001). The demographic characteristics are detailed in Table-I. Topographic parameters are shown in Table-II. The corneal density at the corneal apex was significantly higher in MCD group than in the other groups (p<0.001). The GCD and LCD groups were statistically similar in terms of density of the corneal apex (p=0.079). In MCD group, corneal thickness at the apex and at the thinnest location was significantly thinner, and then in the other groups (p=.002 for thickness at apex between MCD and LCD, and p<.001 for all the remaining comparisons). In MCD group corneal volume was significantly smaller than in the other groups (p<.001 for all comparisons).

## DISCUSSION

Corneal dystrophies were defined by Duke-Elder as “hereditary degenerations of the cornea of unknown etiology occurring bilaterally, manifesting themselves occasionally at birth but more usually during the first or second decades and sometimes later, either stationary or slowly progressive throughout life.^8^ The age of presentation of clinical findings is youngest in MCD, followed by LCD, GCD.^9^ Similarly, the median age in MCD group was lower than the other groups.

In general, corneal dystrophies can be diagnosed by family history and slit lamp biomicroscopy without the need of additional methods. Besides, devices for anterior segment evaluation can be useful during the follow-up or treatment planning.

The Pentacam represent a significant advancement in corneal and anterior segment imaging. The system utilizes two cameras to obtain imaging of the anterior segment.^10^ The cross-sectional images generated by the rotating Scheimpflug camera are used to locate the anterior and posterior corneal surfaces as well as the iris and anterior lens surface.^11^ Thus, the corneal thickness, the density and location of intrastromal opacities, and information about the curvature and elevation of anterior and posterior corneal surfaces can be obtained. The prevention of light penetration to the posterior corneal surface, anterior chamber, iris and lens always must be taken into account during the data interpretation. In order to eliminate as much as possible we included only the maps of patients with acceptable quality specification indices that appear on the Overview Display of the device.

The corneal densitometry is helpful in the following situations: in when deciding the ablation depth during phototherapeutic keratectomy for superficial corneal opacities, or anterior lamellar keratoplasty performed with microkeratome or femtosecond LASER ablation.^12^

In MCD, the opacification extends to the periphery and usually involves the entire thickness of the cornea by the second decade of life.^13^ The opacities of GCD can vary in shape, but are usually grouped into three basic morphologic types: drop-shape, crumb-shaped, and ring shaped. Initially, the stroma between the opacities remains clear.^14^ We evaluated the corneal densities in MCD, GCD and LCD, which demonstrate various corneal opasities with various densities, using the Pentacam densitometry units. Corneal density at the corneal apex in MCD group was significantly higher than in the other groups.

MCD is often associated with reduced central corneal thickness.^15^^-^^17^ In accordance with the literature, we found that corneal thickness at the apex and at the thinnest location is thinner in MCD group than in other groups. Corneal thickness data guides clinician during the treatment planning: PTK, lamellar or penetrating keratoplasty.

**Table-I:** Demographic characteristics of the patients in groups

**Characteristics**	**MCD Group (n=28)**	**GCD Group (n= 20)**	**LCD Group (n=25)**	**p value**
Female/male (n)	13 / 15	12 / 8	13 / 12	.861^*^
Age (years) Median (min, max)	32 (21, 61)	45(19, 68)	53 (42, 68)	<.001&
Right /Left (n)	16 / 12	9 / 11	14 / 11	.793^*^

Chi-Square test, MCD = Macular Corneal Dystrophy,

GCD = Granular Corneal Dystrophy, LCD = Lattice Corneal Dystrophy.

& Oneway-Anova,

**Table-II:** Median values of topography parameters and intergroup comparisons

**Corneal Parameters**	**Median Minimum, Maximum**			**p value** #	**p value** &		
	**MCD Group (n=28 eyes)**	**GCD Group (n=20 eyes)**	**LCD Group (n=25 eyes)**	**All groups**	**MCD vs. GCD**	**MCD vs. LCD**	**LCD vs. GCD**
Density at apex (Pentacam densitometry units)	100 100, 100	68 17, 100	97 34, 100	<.001*	<.001*	<.001*	.079
Thickness (µm)							
at apex	421 344, 541	540 369, 603	459 377, 579	<.001*	<.001*	.002*	.020
at thinnest location	309 234, 470	467 338, 562	405 282, 536	<.001*	<.001*	<.001*	.043
Corneal volume (mm^3^) (3 mm diameter)	43.00 23.65, 65.80	59.60 40.90, 66.20	58.40 49.90, 71.30	<.001*	<.001*	<.001*	.749
Keratometric power deviation (D)	0.60 -5.40, 3.40	1.45 0.20, 2.10	1.80 1.00, 3.30	<.001*	<.001*	<.001*	.003*
K_max_ (D)	51.85 45.80, 62.70	47.65 44.60, 53.00	46.20 43.00, 59.20	<.001*	<.001*	<.001*	.248
Front surface							
K_mean_ (D)	44.60 42.30, 50.90	43.65 41.00, 47.00	40.80 30.80, 44.40	<.001*	.037	<.001*	<.001*
Astigmatism (D)	3.80 1.70, 9.20	2.50 0.30, 6.10	2.30 0.20, 17.50	.169	.063	.206	.680
Q-value (30°)	-0.72 -1.20, 0.10	-0.20 -1.18, 0.19	-0.16 -1.30, 1.94	<.001*	.002*	<.001*	.125
Max. elevation (µm)	30 10, 65	16 2, 30	25 8, 104	.010*	.002*	.538	.051
Back surface							
K_mean_ (D)	-5.20 -10.00, 0.00	-6.15 -6.90, -4.90	-6.40 -7.30, 4.50	.002*	.004*	.001*	.177
Astigmatism (D)	1.70 0.00, 14.40	0.55 0.20, 2.80	0.80 0.10, 14.90	.003*	.002*	.010*	.177
Q-value (30°)	1.15 -2.10, 6.35	-0.15 -0.90, 2.03	-0.50 -0.45, 2.04	<.001*	.001*	<.001*	.042
Max. elevation (µm)	84 23, 407	32 17, 225	52 25, 90	.003*	.012*	.002*	.309
Anterior chamber							
volume (mm^3^)	150. 5 62.0, 447.0	122.0 59.60, 408.0	125 95, 205	.066	.017	<.001*	.748
depth (mm)	2.69 1.08, 7.42	2.31 1.43, 3.94	2.48 1.56, 3.20	.061	.035	.167	.137
angle (degrees)	40.60 28.40, 50.60	36.30 26.90, 49.20	35.20 15.10, 52.70	.288	.038	.101	.775

MCD = Macular Corneal Dystrophy, GCD = Granular Corneal Dystrophy, LCD = Lattice Corneal Dystrophy.

* Statistically significant p values,

# Kruskal Wallis test (p<0.05 significant),

& Mann Whitney U test with Bonferroni correction (p<0.0167),

Corneal volume was significantly smaller in MCD patients as a consequence of thin cornea. K_max_ higher than 48 D, maximum front elevation higher than 15 µm, maximum back elevation higher than 20 µm was accepted as indicator for keratoconus.^18^^,^^19^ These three parameters were significantly higher in MCD group than in other groups. Although literature contains case reports reporting association between keratoconus^20^ and MCD, we think that this has no clinical importance. In conclusion, densitometry on Scheimpflug imaging system gives information on the density of corneal opacities.

## Authors’ Contribution:

**YK and ZAA: **Conceived, designed and did statistical analysis & editing of manuscript.

**YK, AB and FO:** Did data collection and manuscript writing.

## Notes

***Grant Support & Financial Disclosures: ***None.
